# Sirt1 Inhibits Akt2-Mediated Porcine Adipogenesis Potentially by Direct Protein-Protein Interaction

**DOI:** 10.1371/journal.pone.0071576

**Published:** 2013-08-12

**Authors:** Weijun Pang, Yu Wang, Ning Wei, Ruxiang Xu, Yan Xiong, Ping Wang, Qingwu Shen, Gongshe Yang

**Affiliations:** Laboratory of Animal Fat Deposition and Muscle Development, College of Animal Science and Technology, Northwest A&F University, Yangling**,** China; Sun Yat-sen University Medical School, China

## Abstract

Compared with the rodent, the domestic pig is a much better animal model for studying adipogenesis and obesity-related diseases. Currently, the role of Akt2 and Sirt1 in porcine adipogenesis remains elusive. In this study, we defined the effect of Akt2 and Sirt1 on porcine preadipocyte lipogenesis and the regulatory mechanism. First, we found that Akt2 was widely expressed in porcine various tissues and at high level in adipose tissue. Further analysis showed that the expression level of Akt2 was much higher in adipose tissue and adipocytes of the Bamei pig breed (a Chinese indigenous fatty pig) than in that of the Large White pig breed (a Lean type pig), whereas the level of Sirt1 expression was opposite. The expression levels of Sirt1 and Akt2 gradually increased during adipogenic differentiation. Adipogenesis was robustly inhibited in Akt2 deficient fat cells, whereas it was promoted in Sirt1 deficient cells using the lentiviral–mediated shRNA approach. Interestingly, adipogenesis returned to normal in Akt2 and Sirt1 dual–deficient cells, showing that the pro- and anti–adipogenic effects were balanced. Sirt1 inhibited transcriptional activity of Akt2 in a dose-dependent way. Interaction of endogenous Akt2 and Sirt1 was gradually enhanced before day 6 of differentiation, and then attenuated. Akt2 and Sirt1 also interacted with C/EBPα in adipocytes. Moreover, knockdown of Akt2 or/and Sirt1 affected pro–lipogenesis of insulin–stimulated by PI3K/Akt pathway. We further found that Sirt1 respectively interacted with PI3K and GSK3β which were key upstream and downstream components of PI3K/Akt pathway. Based on the above findings, we concluded that the crosstalk between C/EBPα and PI3K/Akt signaling pathways is implicated in Akt2 and Sirt1 regulation of adipogenesis.

## Introduction

Animal models of human diseases have always played a central role in biomedical research for the exploration and development of new therapies. Compared to rodent animals including mice (*Mus musculus*) and rats (*Rattus norvegicus*), pigs (*Sus scrofa*) offer many advantages including close similarity to human physiological characteristic and genetic modulation of fat deposition [1]–[3]. Therefore, the domestic pig is a much better animal model for studying adipogenesis and obese-related diseases. Excessive fat deposition would cause not only bad meat quality but also severe damage to systemic metabolic health. Yet suitable fat, whether in adipose tissue or muscle, contributes importantly to various aspects of meat quality and is central to the nutritional value of meat. In addition, white adipose tissue (WAT) serves as a key regulator for whole–body energy homeostasis [4]. Understanding novel mechanism of genes regulating adipogenesis is helpful to develop strategies for controlling fat deposition of pigs and the treatment of lipid metabolic diseases.

Mouse models suggested that protein kinase B (Akt) and Sirtuin (Sirt) isoforms were involved in the determination of fat mass by interfering with preadipocyte-to-adipocyte transition and regulating lipid storage [5]–[7]. However, little literature can be found about porcine Akt2 and Sirt1 in adipogenesis.

Akt1/2/3 have been well described in literature. They share structural similarities, but differ in expression profiles and functions [8], [9]. Akt1 is the major isoform ubiquitously expressed, while Akt2 is less abundant, except in insulin responsive tissues, such as adipose, muscle and liver tissue [10]. The third isoform Akt3 has been described mostly in brain, testis and beta–cells [11]. Emerging evidence indicates that Akt controls beta-cell proliferation, survival, insulin synthesis and secretion [12], [13]. Importantly, Akt2–null mice exhibit mild growth deficiency, an age–dependent loss of adipose tissue and severe insulin resistance [14], [15]. Experimental as well as clinical studies suggest that Akt2 is a key regulator of adipose tissue mass [16], [17]. Although the function of Akt2 in adipogenesis and fat homeostasis has been extensively studied, no literature reported its role in porcine preadipocyte differentiation.

Sirt1 is a nicotinamide adenine dinucleotide (NAD+) dependent deacetylase [18] which plays important roles in kinds of processes, including stress resistance, energy metabolism and cells differentiation [19]. Compared with wild–type littermates, Sirt1–null mice exhibited a significant reduction in body weight [20]. Up–regulation of Sirt1 is a key to lipolysis and loss of fat in differentiated 3T3–L1 adipocytes [21]. Sirt1 also suppresses adipogenesis in 3T3–L1 cells by inhibiting PPARγ [21]. PPARγ directly interacts with Sirt1 and inhibits Sirt1 activity, forming a negative feedback and self-regulation loop [22]. C/EBPα regulates Sirt1 expression during adipogenesis by directly binding to the Sirt1 promoter [23]. Our previous study indicated that Sirt1 knockdown promoted apoptosis [24] and resveratrol treatment upregulated Sirt1 mRNA, resulting in inhibition of adipogenensis of porcine preadipocytes [25]. However, porcine adipogenic regulation of Sirt1 is still unclear.

Insulin is a key regulator of glucose homeostasis, and its absence is lethal in human cell [26]. Insulin signaling through its receptor tyrosine kinase mediates phosphatidylinositol 3–kinase (PI3K) [27]–[29]. Then, the downstream effectors, Akts, are activated and affect metabolism, proliferation, cell survival, growth and angiogenesis [30]. The signaling is crucial in regulating glucose and lipid metabolism. Some studies suggest that the PI3K/Akt signaling pathway is essential for adipogenesis of human MSCs [31]. IRS-1 knockout mice are lean with a reduced fat cell size and have insulin resistance due to a primary defect of insulin signaling [32]. The adipocytes of the p50α/p55α PI3K knockout mice are smaller than the wild type [33]. Transcriptional activation of PPARγ requires activation of Akt during 3T3–L1 cell differentiation [34]. Sirt1 regulates cancer development by increasing the insulin signaling and mediates interaction with IRS1 [35]. Sirt1 can enhance insulin signaling in part by deacetylating IRS2 [36]. Recent research indicates that Sirt1 modulates PI3K tyrosine phosphorylation and improves muscle insulin sensitivity [37]. Resveratrol induces Sirt1–dependent apoptosis in 3T3–L1 preadipocytes by suppressing AKT activity [38]. However, the function of both Akt2 and Sirt1 in adipocyte biology has not been intensively studied.

Based on above analysis, we investigated the role of Akt2 and Sirt1 in porcine preadipocyte differentiation and the mechanism of them regulating adipogenesis. Collectively, our findings indicate that both Akt2 and Sirt1 play an important role in balancing adipogenesis by C/EBPα and PI3K/Akt signaling pathway. Additionally, the obese pigs and porcine fat cells examined in this study could be a new powerful model to screen novel regulatory genes or drugs for the therapy of human obesity.

## Results

### Expression of Akt2 in Various Porcine Tissues at Different Developmental Stages

**Figure 1:**
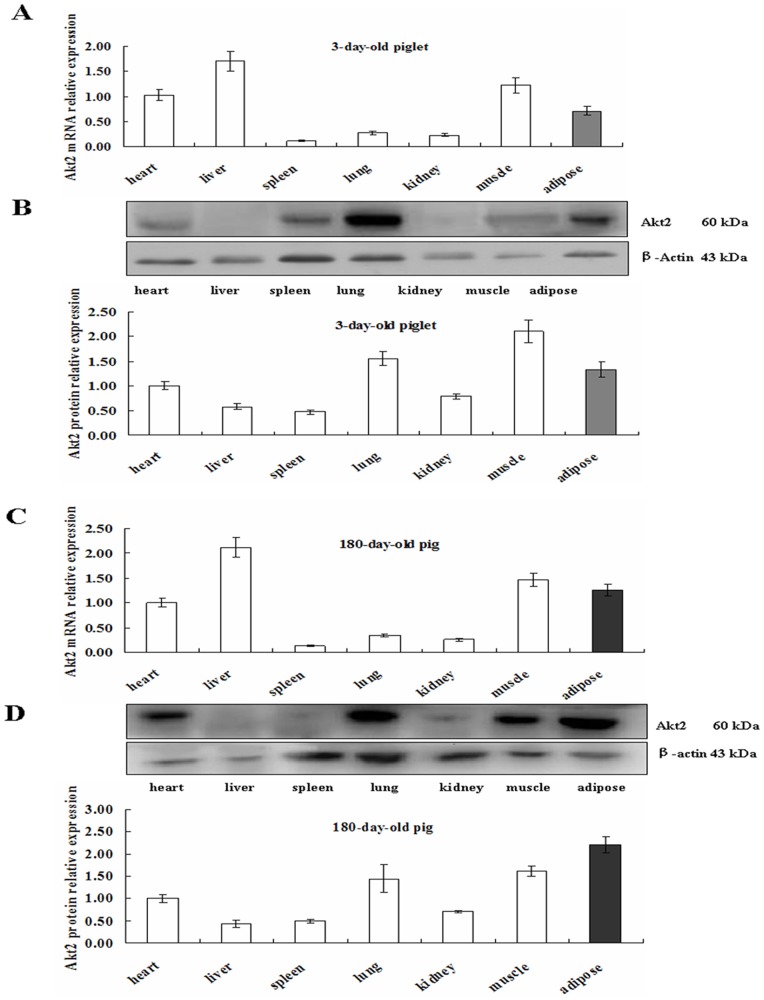
Expression of Akt2 in various tissues from 3–day–old piglets and 180–day–old Landrace×Large White pigs. Total RNA and protein of various tissues were isolated and subjected to qRT–PCR and Western blotting analysis. β–actin is the internal control. To make the figure more appropriate, we selected relative to the levels measured in porcine heart tissue to avoid the bar too high or low. A. Tissue expressional profile abundance of Akt2 mRNA and (B) Akt2 protein at 3–day–old piglets. C. Tissue expressional profile of Akt2 mRNA and (D) Akt2 protein at 180–day–old pigs. The results are represented as means ± SEM of three independent determinations.

Total RNA and protein were extracted from heart, liver, spleen, lung, kidney, muscle and subcutaneous adipose of piglets and adult pigs. Primer sequences for qPCR are shown in Table 1. Akt2 expression was detected by real-time qPCR and Western blot (expressional data of Sirt1 shown in our published paper [25]). The results indicated that Akt2 mRNA and protein were widely expressed in various tissues of 3–day–old piglets (Fig. 1A, B) and 180–day–old adult pigs (Fig. 1C, D). For both stage pigs, Akt2 mRNA was most abundant in heart, liver, muscle and adipose tissue, whereas least abundant in spleen, lung and kidney. The findings showed that Akt2 was widely expressed in various tissues of pigs at developmental stages, speculating that Akt2 may be involved in multiple biological processes in pig and exert different effects in various tissues.

**Table 1:** Primer sequences and parameters for qPCR ampilification of the related genes.

Genes	Oligonucleotides (5′→3′)	GeneBank Accession NO.	Species	Product size/bp
Akt2	F: AAAGTCATCCTGGTGCG	AK347557.1	Sus Scrofa	136
	R: GGGTGCCTGGTGTTCTG			
Sirt1	F: TTGATCTTCTCATTGTTATTGGGTC	DQ868430	Sus Scrofa	62
	R: ACTTGGAATTAGTGCTACTGGTCTTA			
GADPH	F: AAGAGCACGCGAGGAGGA	X9425.1	Sus Scrofa	107
	R: GTCTGGGATGGAAACTGGAAG			
β–actin	F: GCGGCATCCACGAAACTAC	AY550069	Sus Scrofa	177
	R: TGATCTCCTTCTGCATCCTGTC			

### Expression of Akt2 and Sirt1 in Adipose Tissue and Adipocytes of Lean Type Pig and Chinese Indigenous Fatty Pig

**Figure 2:**
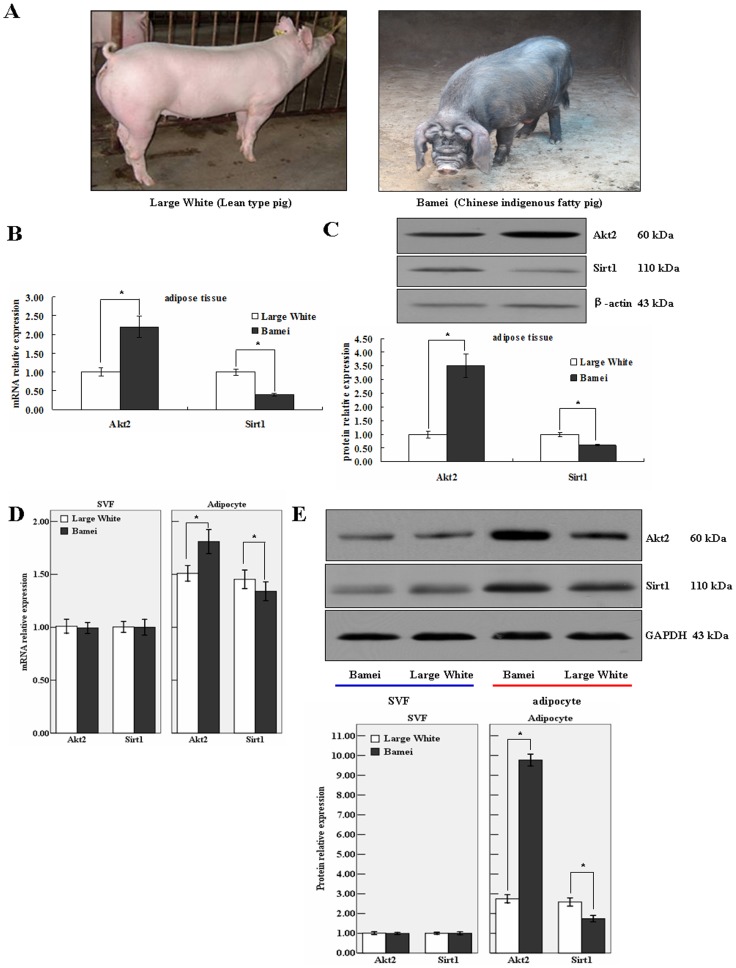
Expression of Akt2 and Sirt1 in adipose tissue and adipocytes extracted from 180–day–old Lean type pigs and Fatty pigs. Total RNA and protein of two type adipose tissues were isolated and subjected to qRT–PCR and Western blotting analysis. β–actin and GAPDH were the internal controls, respectively. A. Large White (Left: Lean type pig) and Bamei (Right: Chinese indigenous fatty pig). B. mRNA relative expression in adipose tissue. C. Protein relative expression in adipose tissue. D. mRNA relative expression in SVF and adipocytes. E. Protein relative expression in SVF and adipocytes.

Bamei pig is a Chinese indigenous fatty pig breed and Large White pig is a lean type pig breed (Fig. 2A). In our experiment, the back fat thickness and fat percentage of Bamei pigs were 2.87 cm and 30.5%, whereas those of Large White pigs were 1.16 cm and 13.4%, respectively (Table 2). The levels of Akt2 mRNA and protein expression were significantly higher in adipose tissue and adipocytes of fatty pigs than that of lean pigs, but the level of Sirt1 expression was significantly lower in fatty pigs when compared to that of lean pigs (Fig. 2B, C, D, E). The interesting novel results suggest that Akt2 and Sirt1 may be implicated in porcine obesity. Moreover, the phylogenetic results showed that the amino acid sequences of Akt2 and Sirt1 between *Sus scrofa* and *Homo Sapiens* were close to each other (Fig. S1A, B). Therefore, physiological functions of *Sus Scrofa* Akt2 and Sirt1 may be similar with that of *Homo Sapiens*, rather than that of *Mus Musculus*.

**Table 2:** Back fat thickness and fat percentage of 180–day–old Large White pig and Bamei pig.

Breed	number	Back fat thickness (cm)	Fat percentage (%)	Lean percentage (%)
Large White	3	1.16±0.12	13.4±0.86	65.5±3.54
Bamei	3	2.87±0.35	30.5±2.12	42.8±2.84

Back fat thickness (cm) = average back fat thickness over three positions  =  (back fat thickness at shoulder+back fat thickness at thorax–waist+back fat thickness at buttock)/3.

Fat percentage (%) = (Fat weight/carcass weight)×100%; Lean percentage (%) = (Lean weight/carcass weight)×100%.

### Time-spatial Expression of Akt2 and Sirt1 during Porcine Preadipocyte Differentiation

**Figure 3:**
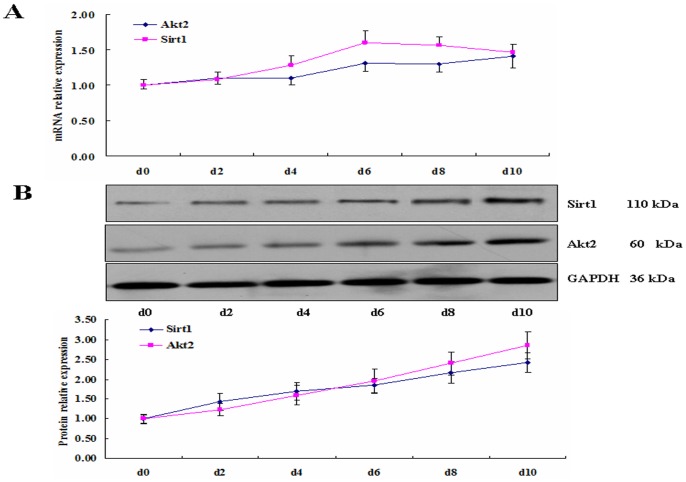
Time course expressions of Akt2 and Sirt1 during porcine preadipocyte differentiation. At the indicated time points during differentiation, porcine fat cells were collected. Total RNA and protein was isolated for analysis of qRT–PCR and Western blot. GAPDH was the internal control. A. mRNA relative expression. B. Protein relative expression.

After confirming the existence of Akt2 and Sirt1 in porcine adipose, we further investigated the time–spatial expression of Akt2 and Sirt1 during preadipocyte differentiation. Sirt1 was reported to suppress the adipogenesis of 3T3–L1 cells and its expression increased during fat cell differentiation [21]. The level of Akt2 protein expression is upregulated during differentiation of SGBS (Simpson-Golabi-Behmel syndrome) preadipocytes [39]. Consisted with these studies, mRNA and protein levels of Akt2 and Sirt1 gradually increased during preadipocyte differentiation (Fig. 3A, B). The results suggest that the increase of Akt2 and Sirt1 protein levels was due to the increase of Akt2 and Sirt1 mRNA levels during differentiation, and the trend of protein and mRNA levels was positive.

### Generation of Akt2 and Sirt1 Deficient Preadipocytes

**Figure 4:**
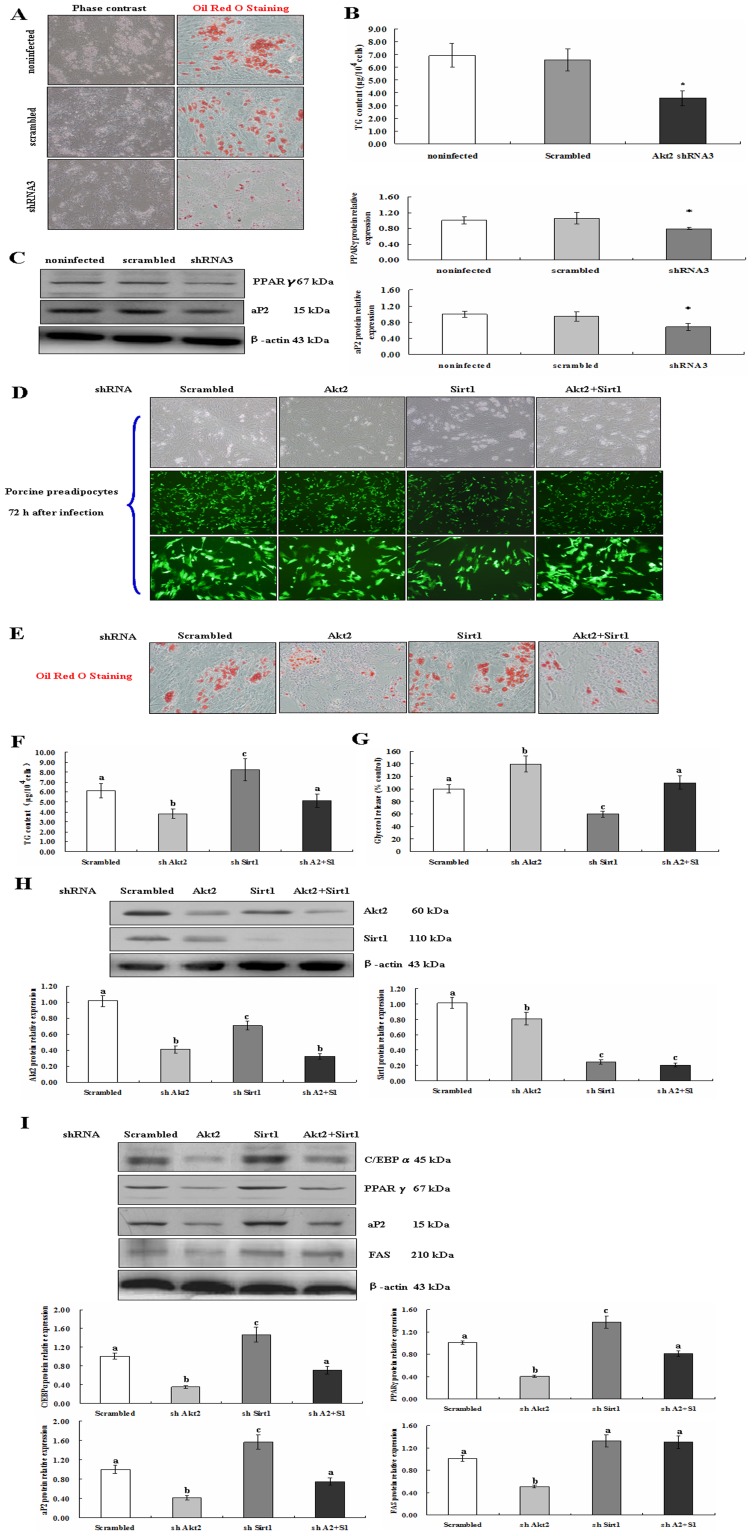
Lipogenesis in both Akt2 and Sirt1 deficient preadipocytes. A. Oil Red O staining (magnification: ×200) and (B) TG content analysis at induced day 8 in Akt2 deficient adipocytes as described in Materials and Methods. C. Expressional downregulation of key adipogenic genes by Akt2 shRNA at induced day 8. D. Porcine preadipocytes at 72 h after infection (Up: ×40, middle: ×40, down: ×200). E. Oil Red O staining at day 8 after induced. Magnification: ×200. F. TG content analysis. G. Glycerol release analysis. H. Akt2 and Sirt1 were downregulated in Akt2 or/and Sirt1 deficient preadipocyte at day 8 after induced. I. Key adipogenic gene expression in Akt2 or/and Sirt1 deficient preadipocytes at day 8 after induced. Data are represented as means ± SEM and from three independent experiments, *P<0.05 and different letter indicates *P*<0.05.

Firstly, we performed renaturation of designed Akt2 sense and antisense shRNA (Fig. S2A; Table S1), and then identified Akt2 shRNA lentivirus vectors by restriction enzyme digestion and sequencing (Fig. S2B, S2C). Similarly, construction and identification of Sirt1 shRNA lentivirus vectors are shown in Fig. S4A–C and Table S2. Therefore, Akt2 and Sirt1 shRNA lentiviral vectors were successfully constructed. HEK293T cells were transfected with lentivirous vector along with packaged vectors (Fig. S3A). Virus supernanant was collected and porcine preadipocytes were infected (Fig. S3B). The results show that the infected efficiency of porcine preadipocytes was up to 85% (Fig. S3B, Fig. 4D).

Consequently, we successfully established porcine preadipocytes stably expressing shRNA constructs specifically targeting Akt2 or Sirt1. The mRNA expression of Akt2 was inhibited by 85% (Fig. S3C). Also, protein expressions of Akt2 or Sirt1 were inhibited by 67% and 62%, respectively (Fig. S3D, Fig. 4H). In addition, Akt2 knockdown did not affect expression of Akt1 (Fig. S5), and Sirt1 knockdown did not affect expression of Sirt2 (Fig. S6).

### Akt2 Knockdown Inhibits whereas Sirt1 Knockdown Promotes Adipogenesis

To further investigate the function of Akt2 and Sirt1 in adipogenesis, porcine preadipocytes were induced using the cocktail method. Under this condition, Oil Red O staining and TG content of the differentiated cells revealed that Akt2-deficient cells accumulate less lipid than control cells (Fig. 4A, B). Moreover, Akt2–deficient cells have lower expression of PPARγ and aP2 (Fig. 4C). In Sirt1–deficient cells, the protein levels of C/EBPα, PPARγ, aP2 and FAS were upregulated and the size of lipid droplets increased (Fig. 4E, I, and Fig. S7). Taken together, these results indicate that Akt2 knockdown inhibited whereas Sirt1 knockdown promoted preadipocyte adipogenesis.

### Adipogenesis Returns to Normal in both Akt2 and Sirt1 Deficient Preadipocytes

Adipogenic differentiation is a very complex process. To explore the effect of both Akt2 and Sirt1 on adipogenesis, the porcine preadipocytes were co–infected by Lenti–shSirt1 and Lenti–shAkt2 (1∶1) and induced to differentiate (Fig. 4D). The cells were staining by Oil Red O (Fig. 4E). TG content (Fig. 4F) and glycerol release (Fig. 4G) were analyzed. The expression levels of C/EBPα, aP2 and FAS were detected using Western blotting. In the co–infected cells, the C/EBPα, PPARγ, aP2 and FAS protein levels were lower and lipid droplets were smaller compared to the cells only infected with Lenti–shSirt1 (Fig. 4H, I and Fig. S7). Interestingly, the findings indicate that pro– and anti–adipogenesis was normal in dual–deficient adipocytes.

### Regulatory Mechanism of Sirt1 and Akt2 during Adipogenesis

**Figure 5:**
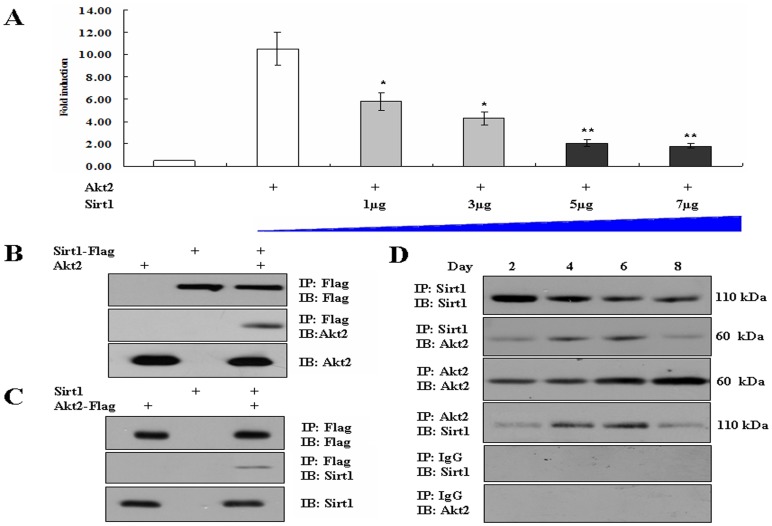
Akt2 interacts with Sirt1 during adipogenesis. A. Sirt1 inhibits transcriptional activity of Akt2 by dose–dependent way. Please reference to Luciferase reporter assays in Materials and Methods. B. Akt2 interacts with Sirt1 in HEK293 cells. HEK293 cells were transfected with Akt2, Sirt1–Flag, or both by the calcium phosphate method. 40 h after transfection, cells were lysed, and Sirt1–Flag proteins were immunoprecipitated with agarose beads and conjugated with anti–Flag antibodies. The precipitated proteins were washed five times and analyzed by Western blotting as indicated. IP: immunoprecipitation. IB: immunoblotting. C. HEK293 cells were transfected with Akt2–Flag, Sirt2, or both by the calcium phosphate method. 40 h after transfection, cells were lysed, and Akt2–Flag proteins were immunoprecipitated with agarose beads and conjugated with anti–Flag antibodies. The precipitated proteins were washed five times and analyzed by Western blotting as indicated. D. Endogenous Akt2 in porcine adipocyte lysate was immunoprecipitated with anti–Akt2 antibody, and coprecipitation of Sirt1 was detected by Western blot assay. Endogenous Sirt1 in porcine adipocyte lysate was immunoprecipitated with anti–Sirt1 antibody, and coprecipitation of Akt2 was detected by Western blot assay. Data were represented as means ± SEM and from three independent experiments, *P<0.05, **P<0.01.

**Figure 6:**
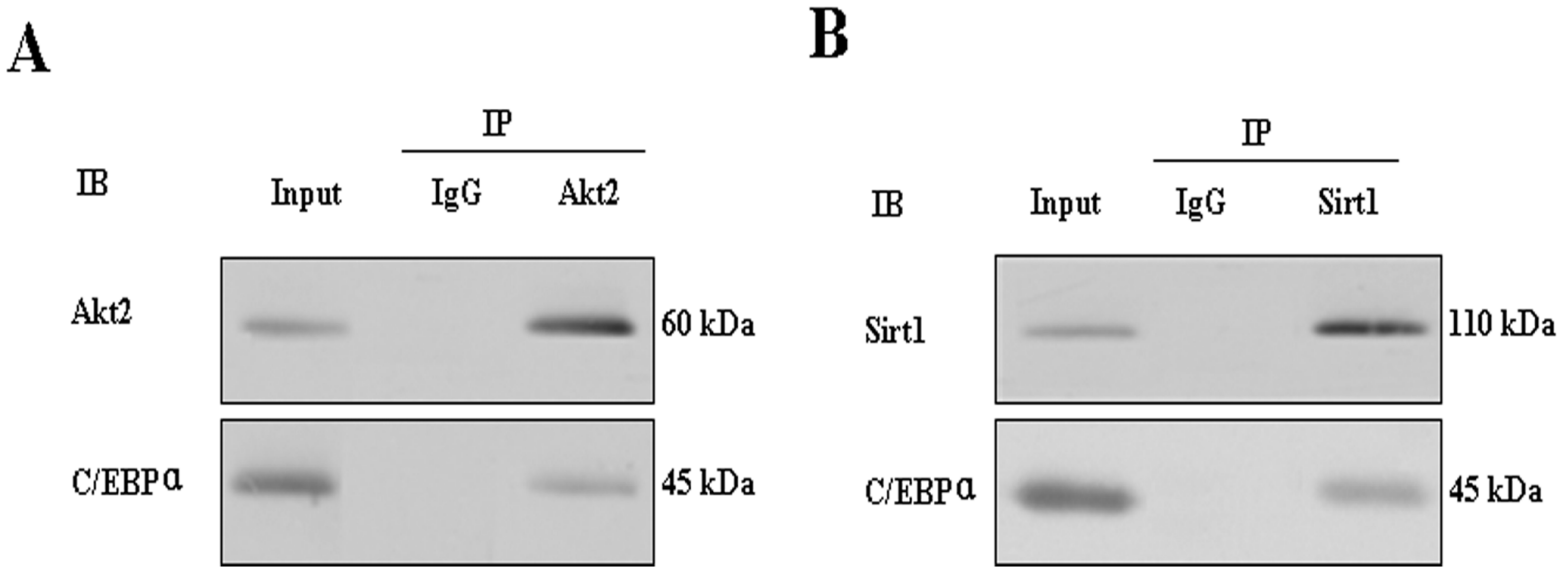
Interaction among C/EBPα, Akt2 and Sirt1 in porcine preadipocytes at day 8 after induced. A. Endogenous Akt2 in fat cell lysate was immunoprecipitated with anti–Akt2 antibody, and coprecipitation of C/EBPα was detected by Western blot assay. B. Endogenous Sirt1 in fat cell lysate was immunoprecipitated with anti–Sirt1 antibody, and coprecipitation of C/EBPα was detected by Western blot assay.

To explore adipogenic mechanism of Sirt1 and Akt2 in preadipocyte differentiation, we performed luciferase reporter assay and immunoprecipitation experiments. Sirt1 inhibits transcriptional activity of Akt2 by dose–dependent way (Fig. 5A). Because of successful examination of the interaction between exogenous Akt2 and Sirt1 in HEK293T (Fig. 5B, C), we further found that endogenic Akt2 and Sirt1 in porcine adipocytes exist protein-protein interaction (Fig. 5D). Interestingly, the binding between Akt2 and Sirt1 was the firmest at day 6 of fat cell differentiation. Akt2 also interacted with C/EBPα, a master gene of preadipocyte differentiation (Fig. 6A), and Sirt1 also interacted with C/EBPα (Fig. 6B).

### Signaling of Key Constituents of PI3K/Akt Pathway in Akt2 or/and Sirt1 Deficient Adipocytes

**Figure 7:**
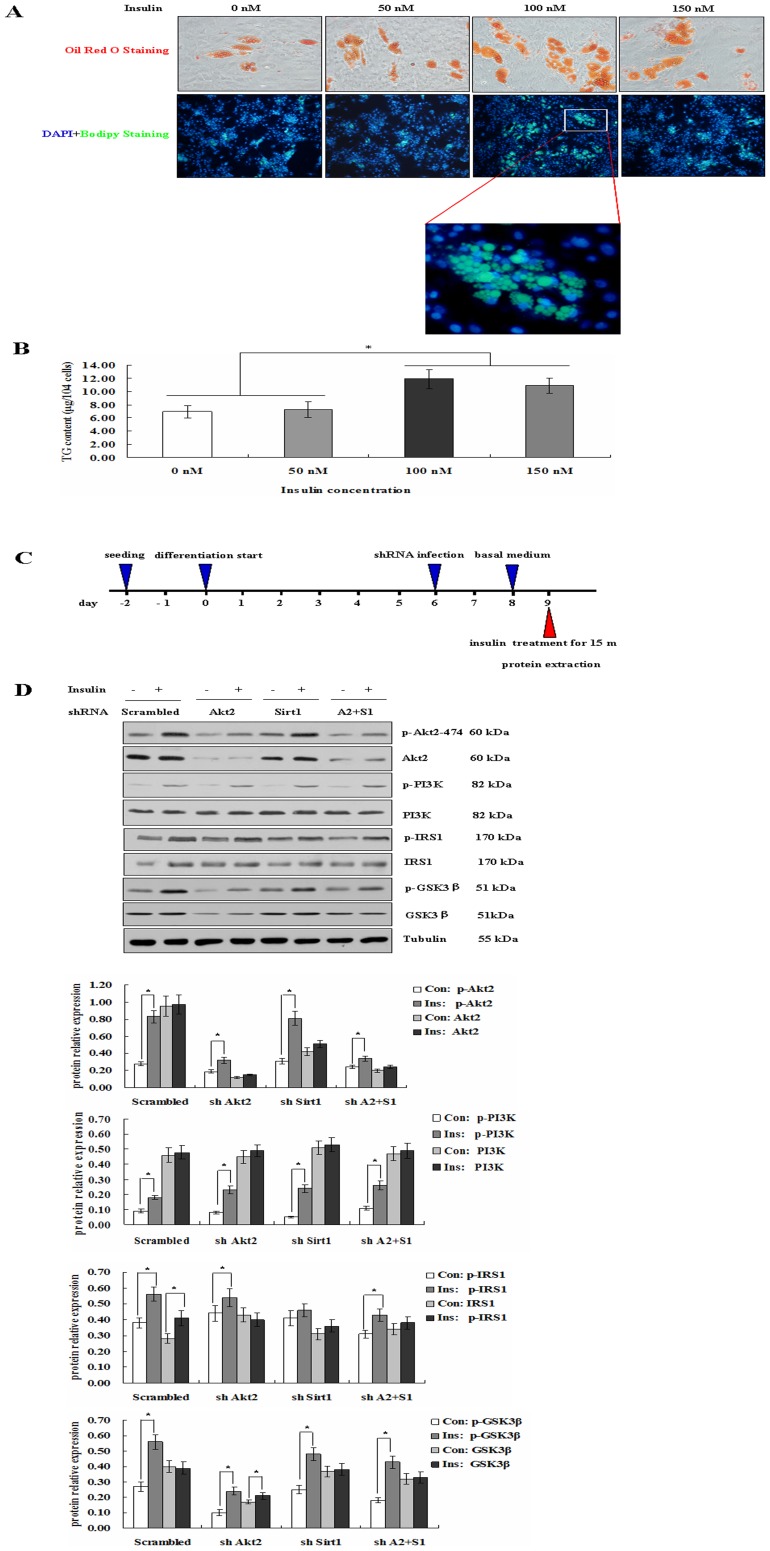
Effect of insulin on target gene activities of PI3K/Akt signaling pathway in Akt2 or/and Sirt1 deficient porcine adipocytes. A. Insulin induces adipogenesis of porcine fat cells. Oil Red O staining (×400) and DAPI+Bodipy staining (×400) to observe the fat cell adipogenesis treated with different insulin concentration (0, 50, 100 and 150 nM) at day 8. B. TG content analysis for cells treated with different insulin concentration at day 8. C. Experimental procedure for insulin treatment 15 minutes of differentiated porcine adipocytes. Preadipocytes induced at day 6 were infected with lentivirus containing Akt2 or/and Sirt1 shRNA. At day 2 after infection, cells were cultured in basal medium without differentiation factors for 24 h, and then stimulated with 100 nM insulin for 15 min. The cells were harvested and protein was extracted for Western blot analysis. D. Regulation of PI3K/Akt signaling in Akt2 or/and Sirt1 deficient fat cells under 100 nM insulin stimulation condition.

To define the effect of insulin on porcine preadipocyte differentiation, the cells were treated with different insulin concentration (0, 50, 100 and 150 nM). By Oil Red O staining, Bodipy staining and TG content assay, we found that 100 nM insulin is the optimal concentration for adipogenesis (Fig. 7A, B).

**Figure 8:**
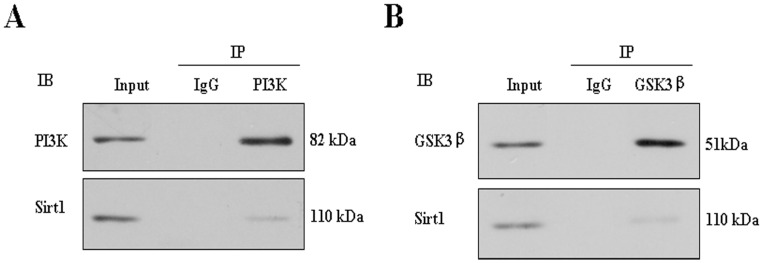
Sirt1 interacts with upstream and downstream genes of PI3K/Akt signaling pathway. At day 8 after induction, porcine fat cells were collected and protein was isolated for immunoprecipitated analysis. A. Sirt1 interacts with PI3K which is an upstream gene in PI3K/Akt signaling pathway. Endogenous PI3K in fat cell lysate was immunoprecipitated with anti–PI3K antibody, and coprecipitation of Sirt1 was detected by Western blot assay. B. Sirt1 interacts with GSK3β which is a downstream gene in PI3K/Akt signaling pathway. Endogenous GSK3β in fat cell lysate was immunoprecipitated with anti–GSK3β antibody, and coprecipitation of Sirt1 was detected by Western blot assay.

**Figure 9:**
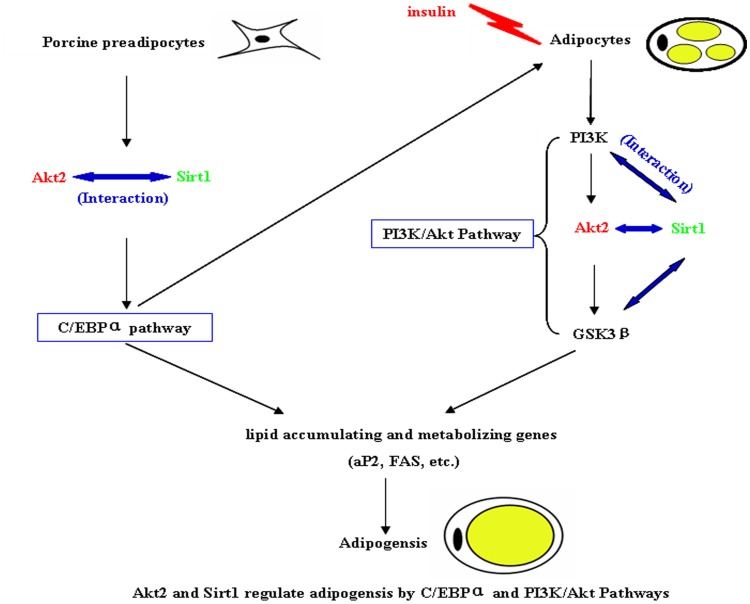
Model for the adipogenic regulation of Akt2 and Sirt1 by C/EBPα and PI3K/Akt signaling pathway.

To further investigate whether PI3K/Akt signaling pathway is involved in regulating adipogenesis of Sirt1 and Akt2, we stimulated the Akt2 or/and Sirt1 deficient adipocytes with 100 nM insulin for 15 min and subjected protein samples to a protein array to study the relevant kinases (Fig. 7C, D). In the Sirt1 deficient adipocytes, phosphorylated IRS1 was altered. As expected, IRS1, PI3K and their phosphorylation did not changed in Akt2 deficient adipocytes, whereas GSK3β and its phosphorylation significantly decreased in Akt2 deficient adipocytes. Interestingly, GSK3β and its phosphorylation were higher in the dual–deficient adipocytes than in the Akt2 deficient adipocytes, but lower than in Sirt1–deficient adipocytes. This suggested that Akt2 played a direct central role, but Sirt1 might play indirect role in insulin–stimulated adipocyte adipogenesis through PI3K/Akt pathway. To further explore the indirect role, we performed IP experiment of Sirt1 with PI3K and GSK3β. The results showed that Sirt1 interacted with PI3K which is upstream of PI3K/Akt pathway (Fig. 8A, Fig. S8), and also interacted with GSK3β which is upstream of PI3K/Akt pathway (Fig. 8B). Based on above findings, Akt2 and Sirt1 regulated adipogensis by PI3K/Akt pathway (Fig. 9).

## Discussion

Many recently published data about Akt2 and Sirt1 expression were obtained using mouse model [40]–[43]. Here, by analysis of tissue expression, to our surprise, we newly found that Akt2 mRNA and protein expression did not match in liver and lung. Similar phenomenon has been reported for other genes [44], [45]. One of the reasons may be a potential mechanism of miRNA regulation, such as miR–184 [46] and miR–203 [47] which directly targets and leads to degradation of Akt2. Whether these microRNAs are present in liver and lung and whether these microRNAs are responsible for the different pattern of Akt2 mRNA and protein expression need to be explored in the future.

Interestingly, the levels of Akt2 mRNA and protein are much higher in both adipose tissue and adipocyte of Bamei pig than that of Large White pig, whereas the level of Sirt1 expression is right opposite. Akt2 and Sirt1 therefore may be involved in formation of porcine obesity. Because of the similar anatomy characteristic between human and pig, it is needed to further measure some physiological parameters, such as blood glucose levels and insulin resistance, in fatty and lean pigs in future. Therefore, it is necessary to develop a porcine obesity model for further studies.

Our previous study found that in porcine preadipocytes the efficiency of direct transfection with vector was much low, just to 20%. To improve the efficiency, we developed lentivirus–mediated method. Our results indicated that the infected efficiency is up to 85% with either single shRNA or two shRNAs using lentivirus–mediated approach. The infected method provides a novel and valid way to study regulatory adipogenic function of Akt2, Sirt1 and other genes in porcine preadipocytes. Moreover, consisted with recent results of 3T3–L1 cells [48], [49], Akt2 knockdown significantly inhibited, whereas Sirt1 knockdown promoted porcine preadipocyte differentiation. However, a meaningful problem appears how preadipocytes balance function of positive and negative adipogenic genes during differentiation.

Many factors participate in the up- or down-regulation of adipogenesis. Among these factors, PPARγ and C/EBP family of transcription factors play a central role. In addition to PPARγ and C/EBP family of transcription factors, Akt2 is also a positive regulator [39]. Conversely, many factors suppress adipogenesis. Sirt1 is one of those negative regulators [21]. To understand the regulatory adipogenic effect of both Akt2 and Sirt1, we co–infected porcine preadipocytes using 1∶1 lentivirous containing shAkt2 and shSirt1. The results indicated that adipogenesis was inhibited. The findings hint that the pro-adipogenesis effect of Sirt1 knockdown was partially repressed by Akt2 knockdown. It has been reported that C/EBPα regulates Sirt1 expression during adipogenesis by directly binding to the Sirt1 promoter [50]. Here, by IP analysis we further found that both endogenous Akt2 and Sirt1 interacted with C/EBPα in porcine adipocytes. It indicated that Akt2 and Sirt1 also regulated adipogenesis by interaction with C/EBPα.

To explain how to inter-regulate between Akt2 and Sirt1, we performed IP experiment of the two proteins. We found that exogenous Akt2–Sirt1 interaction existed in HEK293T cells of ectopic over–expression, and then endogenous protein-protein interaction was confirmed during preadipocyte differentiation. Interestingly, the ability of bindings was the firmest on day 6 during differentiation. Based on these findings, it can be speculated that the interaction between Akt2 and Sirt1 is involved in adipogenesis.

Several studies indicate that PI3K/Akt signaling pathway plays a key role in regulating preadipocyte differentiation and adipogenesis in vitro [51], [52]. A key contributor of adipose tissue mass regulation is Akt, which is part of the intracellular signaling cascade activated by insulin [53]. In spite of a close homology of Akt1–3, the regulation of adipose tissue mass is isoform specific [9], [53], [54]. Adipose tissue homeostasis involves the generation of new adipocytes from preadipocytes and the elimination of old adipocytes. Fat mass is predominantly determined by the volume of adipocytes, which is regulated by competing processes of lipogenesis and lipolysis [55]. Insulin is an important extracellular regulator of both adipocyte number and function. In order to further investigate whether PI3K/Akt signaling pathway involved in porcine adipogenic differentiation of Sirt1 and Akt2, we stimulated the Akt2 or/and Sirt1 deficient adipocytes with 100 nM insulin for 15 min and subjected protein samples to a protein array to study the up– or down–stream kinases. As expected, Akt2 and p–Akt2 were decreased in Akt2 or both gene dificient adipocytes. Interestingly, in Akt2 or/and Sirt1 deficient adipocytes the levels of PI3K, p-PI3K, IRS1 and p–IRS1 were not changed, whereas the levels of GSK3β and p–GSK3β was downregulated in Akt2 or both gene-deficient adipocytes. The results suggest that Akt2 plays a central role in adipogenic process through PI3K/Akt pathway. We further found that Sirt1 interacts with PI3K and GSK3β at day 8 after preadipocye differentiation. It is shown that adipogenic effect of both gene–deficient adipocytes involves the interaction of Sirt1 with key upstream and downstream components of PI3K/Akt signaling pathway. Moreover, we need point out that this is likely the case in the model (Fig. 9), although no experiments address that Akt2 and Sirt1 shRNA affects adipogenesis through C/EBPα of GSK3β.

In conclusion, our findings demonstrated that adipogenesis of porcine preadipocytes was balanced by positive regulation of Akt2 and negative regulation of Sirt1 through crosstalk between C/EBPα and insulin–stimulated PI3K/Akt pathways.

## Materials and Methods

### Ethics Statement

All study involving pigs were conducted according to the regulation approved by the Standing Committee of Shaanxi People's Congress, P. R. China. Sample collection was approved by the ethics committee of Northwest A&F University. The entire experimental procedure was approved and supervised by the Animal Care Commission of the College of Veterinary Medicine, Northwest A&F University.

### Experimental Pigs and Sample Collection

Six healthy male crossbred (Landrace×Large White) piglets (3–day–old), six male crossbred (Large White×Landrace) adult pigs (180-day-old), three Large White pigs (180–day–old) and three Bamei pigs (180–day–old) were provided by the Northwest A&F University experiment station (Yangling, China). Animals were allowed access to feed and water ad libitum under same normal conditions and were humanely sacrificed as necessary to ameliorate suffering. All tissue sample collection was approved by the ethics committee of Northwest A&F University. Heart, liver, lung, kidney, spleen, muscle and subcutaneous adipose were isolated, collected, quickly frozen in liquid nitrogen, and then kept at –80°C until subsequent analysis.

### Reagents

Dulbecco’s modified Eagle’s medium (DMEM)/F12 and fetal bovine serum (FBS) were purchased from Hyclone (America). CaCl_2_·2H_2_O, NaH_2_PO_4_·H_2_O, NaCl, Hepes, Polybrene, insulin, dexamethasone, IBMX, Oil red O and anti-Flag M2 agarose affinity gel were purchased from Sigma (USA). Bodipy was purchased from Invitrogen (America). TRIzol reagent and Real Time reagent Kit were purchased from TaKaRa Biotechnology (Dalian, China). The glycerol assay kit was from Randox Crumlin Co. (United Kingdom). The dual luciferase kit was from Promega (Beijing, China). The following antibodies were used: IRS1, PI3K, phospho–PI3K (Millipore, USA), Akt1 (1∶1000), Akt2 (1∶1000), phospho–Akt2–Ser474 (1∶1000) (Bioworld, USA), Sirt1 (1∶1000), Sirt2 (1∶1000), C/EBPα (1∶1000), PPARγ (1∶500), GAPDH (1∶5000), β–actin (1∶1000) and Tubulin (1∶5000) (Santa Cruz Biotechnology, Santa Cruz, USA), FAS (1∶1000), aP2 (1∶1000), GSK3β (1∶1000), phospho-GSK3β (1∶1000) (Cell Signaling Technology, USA). In addition, anti–Flag (1∶2000) and anti-rabbit or mouse secondary antibody conjugated with horseradish peroxidase (1∶3000) were purchased from Santa Cruz Biotechnology (USA). Goat anti–rabbit IgG and goat anti-mouse IgG magnetic beads were from Polysciences (USA).

### Isolation of SV-fraction and Adipocytes

SV-fraction and adipocytes were isolated from white adipose tissue of Bamei and Large White pigs. Briefly, subcutaneous fat pads from 6-month-old Bamei and large white pigs were minced in Krebs-Ringer phosphate buffer and digested with 1 mg/ml collagenase type I at 37°C for 1h, and then digested tissue was filtered through a nylon mesh and centrifuged at 500 rpm for 10 min. The top layer (adipocyte fraction) was collected. The remaining was centrifuged again at 1,500 rpm for 10 min, and the pellet (stromal-vascular fraction, SVF) was collected. Protein and RNA were extracted from both fractions for qPCR and western blot analysis.

### Porcine Preadipocyte Culture

Subcutaneous adipose tissue was removed from the neck of piglets (day 1–3) under sterile conditions, and porcine preadipocytes were isolated by the previously described method [22], [23]. The induced method is similar to that of human preadipocytes [56]. In brief, porcine preadipocytes were cultured to confluence in DMEM supplemented with 10% (v/v) calf serum. At 2 days post-confluence (designated day 0), cells were induced to differentiate with DMEM supplemented with 10% (v/v) FBS, 1 µM dexamethasone, 0.5 mM isobutylmethylxanthine, 1 µg/ml insulin for 2 days, then every 2 days, media was changed with DMEM supplemented with 10% (v/v) FBS and 1 µg/ml insulin.

### Oil Red O and Bodipy Staining

Harvested porcine primary adipocytes were washed three times with PBS and fixed with 10% formaldehyde for 30 min at room temperature. After washing three times with PBS, cells were stained with 1% filtered Oil Red O for 30 min or Bodipy for 15 min at room temperature. Then, Oil Red O or Bodipy solution was removed, wash adipocytes three times with PBS. The end, adipocytes were examined with microscopy. Additionally, after Oil red O staining shown, 6 visual fields per well was randomly selected and statistical analysis of the size of lipids droplet was implemented using Image-Pro Plus 6.0 image analysis software.

### Triglyceride Content Assay

Triglyceride (TG) content analysis was conducted according to the previous method [57]. Briefly, cultured cells on six-well plates were washed twice with PBS, scraped off into 0.4 ml of 25 mM Tris–HCl (pH 7.5) containing 1 mM EDTA, and then homogenized. TG in the cell lysate was extracted with the same volume of chloroform–methanol (2∶1, v/v) and quantified enzymatically using a TG Test Kit.

### Lipolysis Assay

Porcine Akt2 or/and Sirt1 deficient preadipocytes were stimulated for differentiation in high-glucose medium for 8 d. Glycerol content in the medium was measured by the glycerol assay kit following the manufacturer's instructions. Briefly, 10 µl supernatant of culture was incubated with 1 ml assay reagent for 5 min at 37°C. The absorbance was measured by spectrometer at 520 nm.

### RNA Preparation and Real-time Expression Analysis

Porcine adipocyte lysates were homogenized and extracted for total RNA using TRIzol reagent by standard techniques. The integrity of RNA was checked on 2% agarose gel, and total RNA concentration was estimated by a spectrophotometer (Thermo). 2 µg of total RNA was reverse–transcribed to synthesize cDNA using the PrimeScript RT–reagent kit for real–time PCR after treatment with DNAse I to remove contaminating genomic DNA. We designed specific Akt2 primer for PCR and its sequence was shown Table 1. Real–time PCR amplification reactions were carried out on a Bio-Rad iQ5 by SYBR Premix Ex TaqTMII chemistry detection under amplification conditions. Quantification of the mRNA data was done using the comparative threshold cycle method, with the modification that the relative efficiency of each primer was included in the calculation. The specificity of the PCR amplification was always verified with melting curve analysis.

### Western Blotting Analysis

The cellular protein was extracted in the lysis buffer (pH7.5) containing 50 mM Tris–HCl, 0.5% Triton X–100, 2 mM EDTA, 150 mM NaCl, and 1 mM PMSF. The protein content was measured by Peterson’s method. The extracted protein was electrophoresed in 10% SDS–polyacrylamide gel under reducing conditions, and electrotransfered to nitrocellulose membranes. After blocking in 5% de–fatted milk, the membranes were incubated with primary antibodies overnight at 4°C. After washing three times with Tris-buffered saline/0.1% Tween 20 (TBST) at room temperature, the membranes were hybridized with secondary antibody for 1 h at room temperature, and again washed three times with TBST. The targeted proteins were detected using the Gel Doc XR System as per the instructions of the manufacturer.

### Luciferase Reporter Assays

A luciferase reporter construct under the direction of a 200-bp fragment from human complement component C3 promoter has a high–affinity Akt2–binding site. Reporter construct (1 µg) was transfected into HEK293T cells along with pcDNA–Sirt1 using the calcium phosphate method. Renilla luciferase reporter plasmid (pRL–TK) was included as an internal control for transfection efficiency. After transfection (24 h), cells were lysed, and luciferase activity was measured with the dual luciferase kit according to the manufacturer’s instruction. Brieﬂy, 20 µl of cell lysate was mixed with 90 µl LAR II reagent, the fluorescence was recorded as firefly luciferase activity, and then 75 µl of stop and glow buffer was added to measure the Renilla luciferase. Firefly luciferase activity was normalized to Renilla luciferase activity.

### Immunoprecipitation

Cell lysate 200 µl was supplemented with 300 µl lysis buffer to get the appropriate protein concentration and volume. 40 µl of anti-Flag M2 agarose affinity gel were added to the lysate and rocked in 4°C overnight. After washing for 5 times with lysis buffer, protein was eluted with 2×loading buffer for Western blot analysis. For endogenous protein–protein interaction, 10 mg total porcine adipocyte lysates were incubated with 4 µg antibodies against Akt2, Sirt1, C/EBPα, PI3K, or GSK3β overnight and then captured by 1 ml goat anti-rabbit or goat anti–mouse IgG magnetic beads for 4 h. After washing 4 times with lysis buffer, proteins were eluted with 2×loading buffer for Western blot analysis.

### Statistical Analysis

All data were obtained from at least three independent experiments. Values were expressed as means ± SEM. Statistics were calculated with SPSS statistics v13.0 software. Student’s *t* test was used for individual comparisons. Multiple comparisons were assessed by one-way ANOVA followed by Dunnett’s tests. Difference between groups were considered statistically significant if *P*<0.05.

## Supporting Information

Figure S1 **Amino acid sequence phylogenetic analysis of Akt2 and Sirt1 proteins.** A.Phylogenetic tree of the Akt2 protein. The phylogenetic tree is constructed by the Neighbor-Joining method. The numbers by the branches indicate bootstrap values based on 1000 replications. Branch lengths are relative to the degree of divergence. Akt2 protein Accession No.: *Sus Scrofa* (NP_001243708.1), *Homo Sapiens* isoform 1–3 (NP_001617.1, NP_001229956.1 and NP_001229957.1), *Mus Musculus* isoform 1 and 2 (NP_001103678.1 and NP_031460.1), *Danio rerio* (NP_937789.1), *Bos taurus* (NP_001193075.1). B. Phylogenetic tree of the Sirt1 protein. Sirt1 protein Accession No.: *Sus Scrofa* (NP_001139222.1), *Homo Sapiens* (NP_036370.2), *Mus Musculus* (AAR23928.1), *Gallus gallus* (NP_001004767.1), *Xenopus laevis* (NP_001136381.1), and *Drosophila melanogaster* (NP_477351.1).
(TIF)

Figure S2 **Construction and identification of Akt2 shRNA lentiviral vectors.** A. Examining of Akt2 double stand shRNA forming by agarose gel electrophoresis. B, C. Identification of Akt2 positive lentiviral vector by restriction enzyme digestion. D. Identification of Akt2 positive lentiviral vector by DNA sequencing.
(TIF)

Figure S3 **Detection of Akt2 knockdown in adipocytes.** A. HEK293T at 48 h after transfection. Magnification (up: ×40, down: ×40). B. Porcine preadipocytes at 72 h after infection (up: ×40, down: ×200). C. Akt2 knockdown by RNAi insignificantly downregulated its mRNA and (D) protein expression.
(TIF)

Figure S4 **Construction and identification of Sirt1 shRNA lentiviral vectors.** A. Examining of Sirt1 double stand shRNA forming by agarose gel electrophoresis. B. Identification of Sirt1 positive lentiviral vector by restriction enzyme digestion. C. Identification of Sirt1 positive lentiviral vector by DNA sequencing.
(TIF)

Figure S5 **Akt2 knockdown doesn’t affect expression of Akt1 in porcine preadipocytes.**
(TIF)

Figure S6 **Sirt1 knockdown significantly inhibits its expression but doesn’t affect expression of Sirt2 in porcine preadipocytes.**
(TIF)

Figure S7 **Knockdown of Akt2 and Sirt1 affects lipid droplet size.** A. Oil Red O staining at day 8 after infection. Bar, 100 µm or magnification: ×200. B. Range of the lipid droplet size (µm). C. Average value of lipid droplet size (µm). Different letter indicates *P*<0.05.
(TIF)

Figure S8 **Sirt1 does not interact with Tubulin in adipocytes.** At day 8 after induction, porcine fat cells were collected and protein was isolated for immunoprecipitated analysis. A. Sirt1 does not interact with Tubulin. Endogenous Sirt1 in fat cell lysate was immunoprecipitated with anti–Sirt1 antibody, and coprecipitation of Sirt1 was detected by Western blot assay. B. Tubulin does not interact with Sirt1. Endogenous Tubulin in fat cell lysate was immunoprecipitated with anti–Tubulin antibody, and coprecipitation of Tubulin was detected by Western blot assay.
(TIF)

Table S1 **Porcine Akt2 shRNA sense strands and antisense strands.**
(DOC)

Table S2 **Porcine Sirt1 shRNAs sense strands and antisense strands.**
(DOC)
