# Supplementary figures and images for: Sirt1 Inhibits Akt2-Mediated Porcine Adipogenesis Potentially by Direct Protein-Protein Interaction
Source: PLoS One. 2013 Aug 12;8(8):e71576. doi: 10.1371/journal.pone.0071576 (PMC3741135; doi:10.1371/journal.pone.0071576)

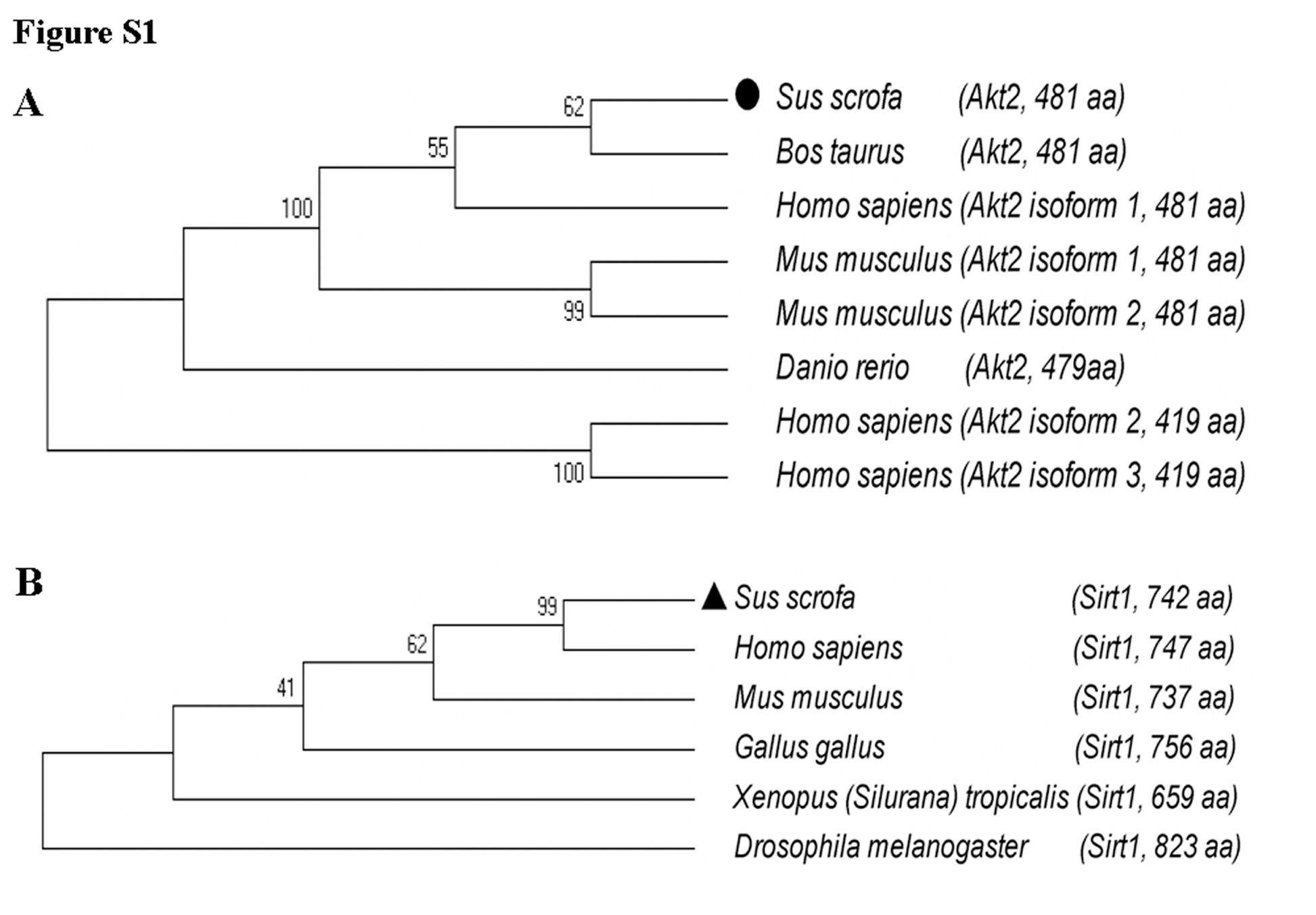

Supplement: Figure S1 — Amino acid sequence phylogenetic analysis of Akt2 and Sirt1 proteins. A.Phylogenetic tree of the Akt2 protein. The phylogenetic tree is constructed by the Neighbor-Joining method. The numbers by the branches indicate bootstrap values based on 1000 replications. Branch lengths are relative to the degree of divergence. Akt2 protein Accession No.: Sus Scrofa (NP_001243708.1), Homo Sapiens isoform 1–3 (NP_001617.1, NP_001229956.1 and NP_001229957.1), Mus Musculus isoform 1 and 2 (NP_001103678.1 and NP_031460.1), Danio rerio (NP_937789.1), Bos taurus (NP_001193075.1). B. Phylogenetic tree of the Sirt1 protein. Sirt1 protein Accession No.: Sus Scrofa (NP_001139222.1), Homo Sapiens (NP_036370.2), Mus Musculus (AAR23928.1), Gallus gallus (NP_001004767.1), Xenopus laevis (NP_001136381.1), and Drosophila melanogaster (NP_477351.1). (TIF) [file pone.0071576.s001.tif]

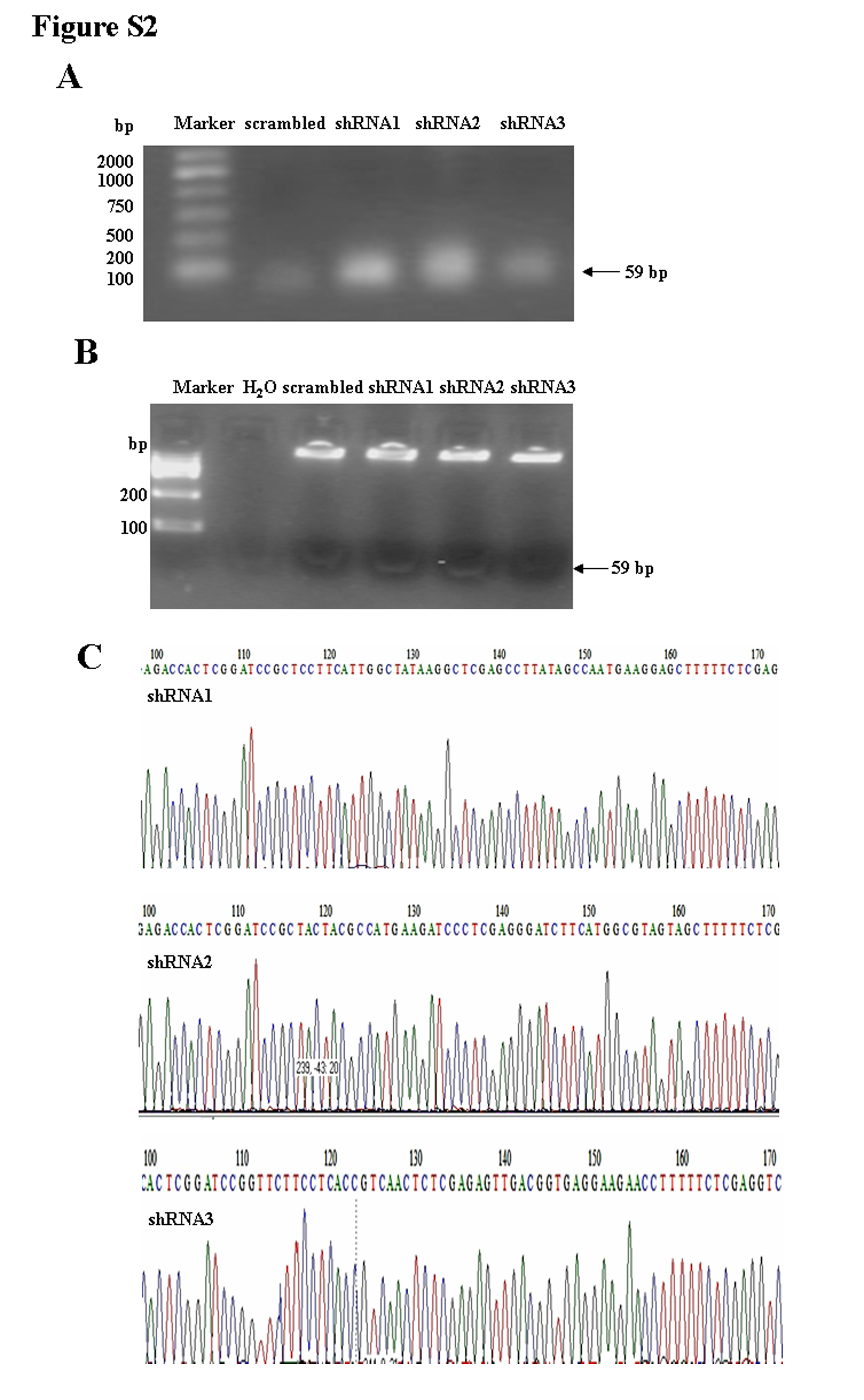

Supplement: Figure S2 — Construction and identification of Akt2 shRNA lentiviral vectors. A. Examining of Akt2 double stand shRNA forming by agarose gel electrophoresis. B, C. Identification of Akt2 positive lentiviral vector by restriction enzyme digestion. D. Identification of Akt2 positive lentiviral vector by DNA sequencing. (TIF) [file pone.0071576.s002.tif]

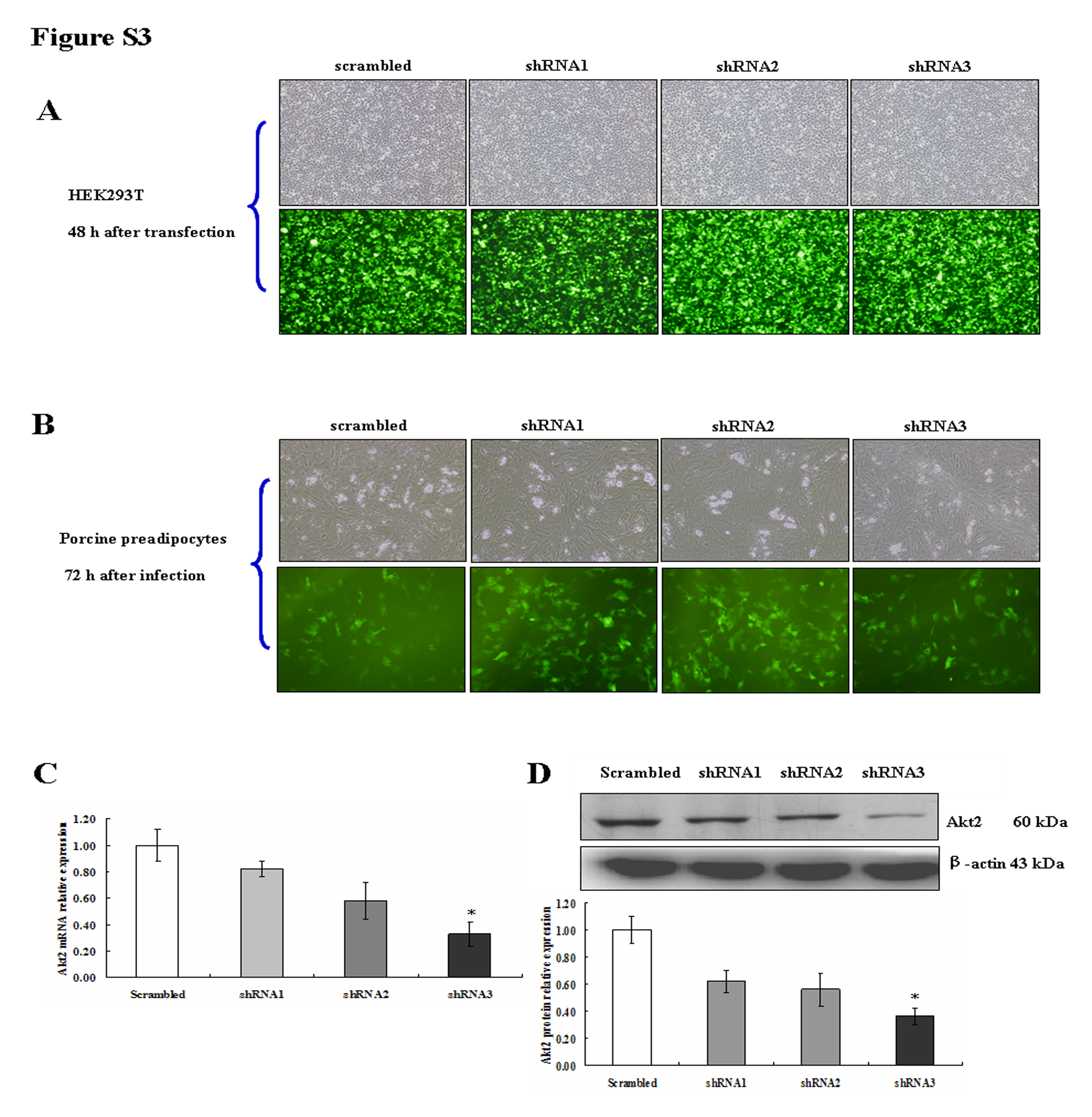

Supplement: Figure S3 — Detection of Akt2 knockdown in adipocytes. A. HEK293T at 48 h after transfection. Magnification (up: ×40, down: ×40). B. Porcine preadipocytes at 72 h after infection (up: ×40, down: ×200). C. Akt2 knockdown by RNAi insignificantly downregulated its mRNA and (D) protein expression. (TIF) [file pone.0071576.s003.tif]

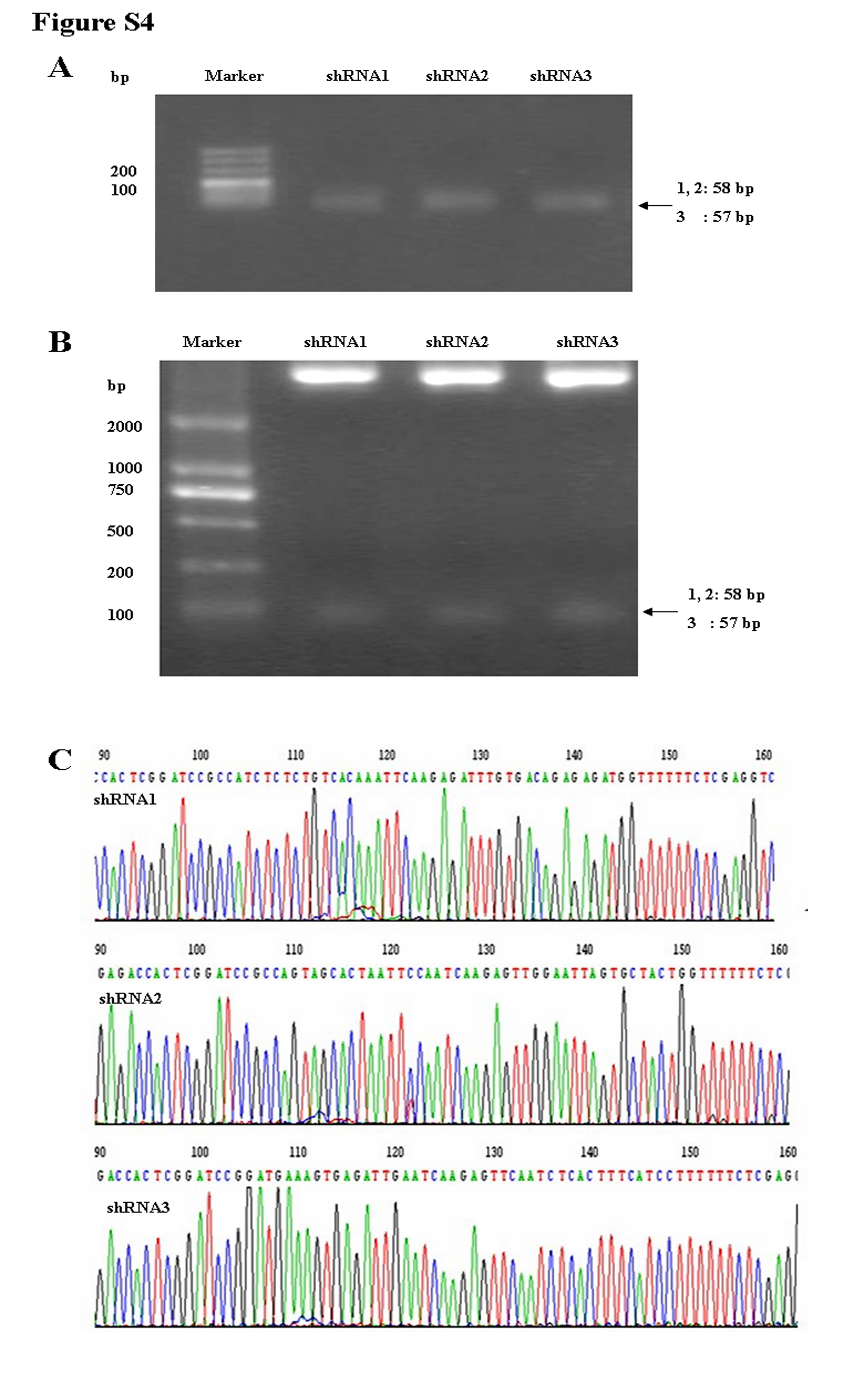

Supplement: Figure S4 — Construction and identification of Sirt1 shRNA lentiviral vectors. A. Examining of Sirt1 double stand shRNA forming by agarose gel electrophoresis. B. Identification of Sirt1 positive lentiviral vector by restriction enzyme digestion. C. Identification of Sirt1 positive lentiviral vector by DNA sequencing. (TIF) [file pone.0071576.s004.tif]

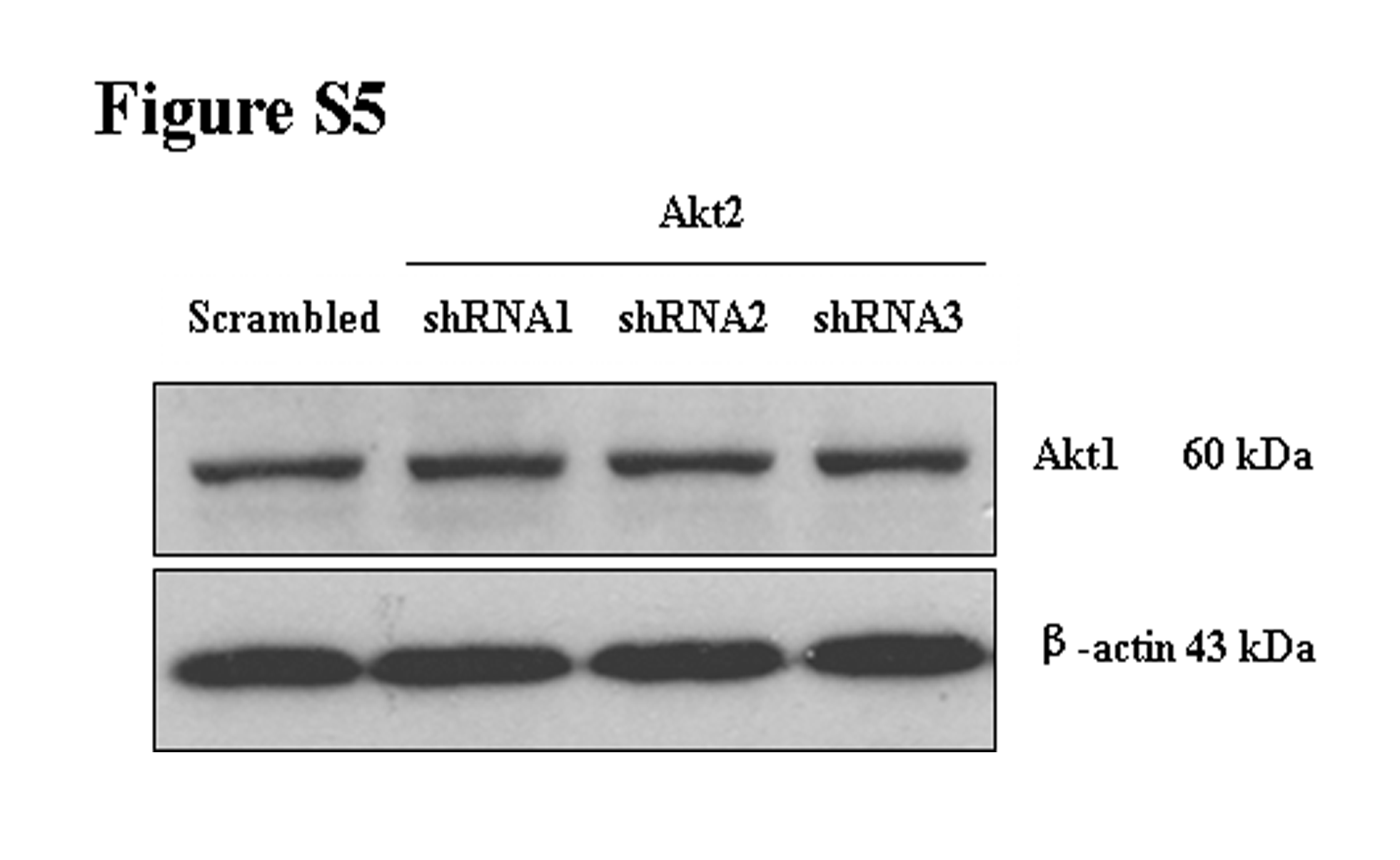

Supplement: Figure S5 — Akt2 knockdown doesn’t affect expression of Akt1 in porcine preadipocytes. (TIF) [file pone.0071576.s005.tif]

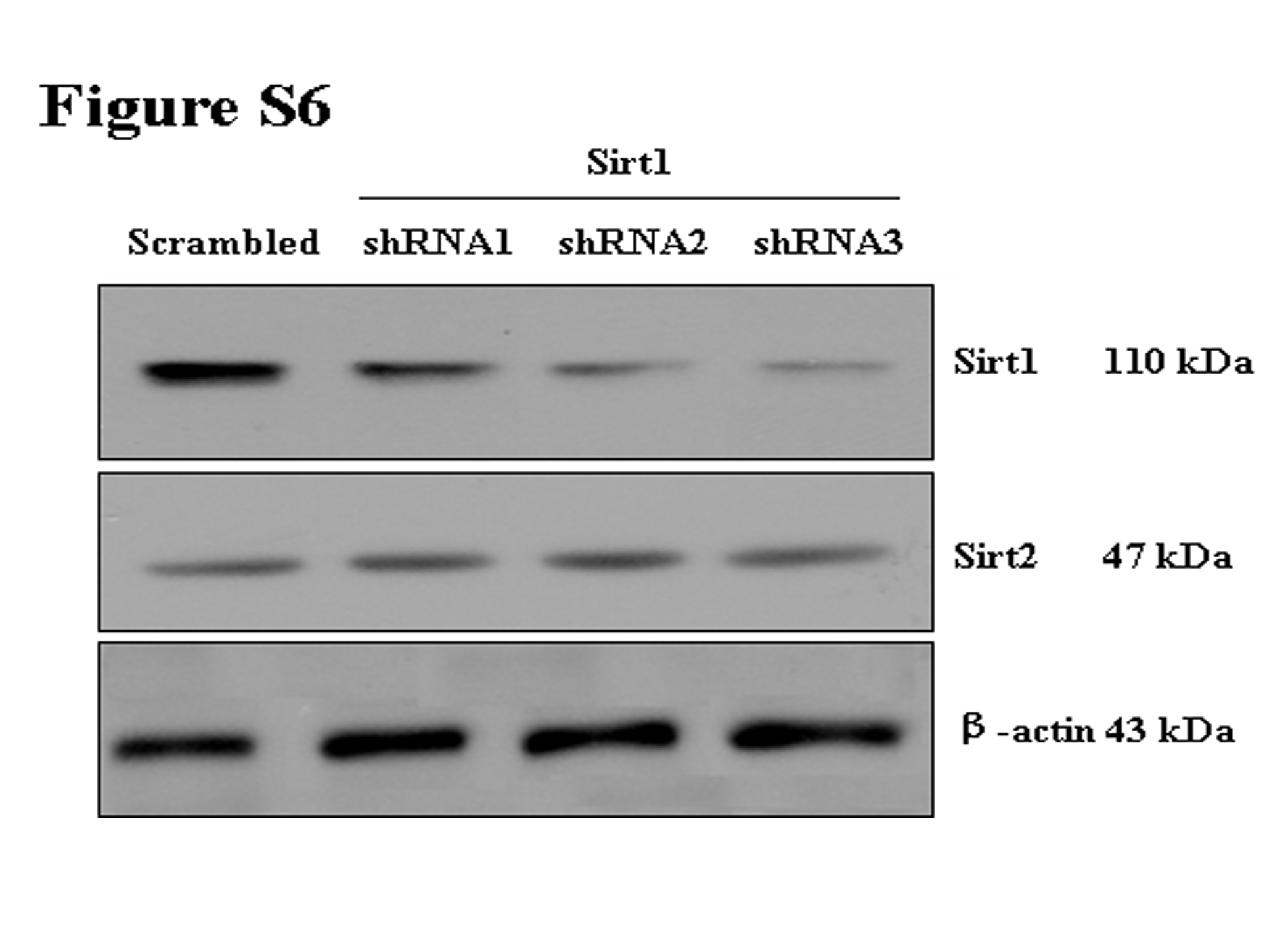

Supplement: Figure S6 — Sirt1 knockdown significantly inhibits its expression but doesn’t affect expression of Sirt2 in porcine preadipocytes. (TIF) [file pone.0071576.s006.tif]

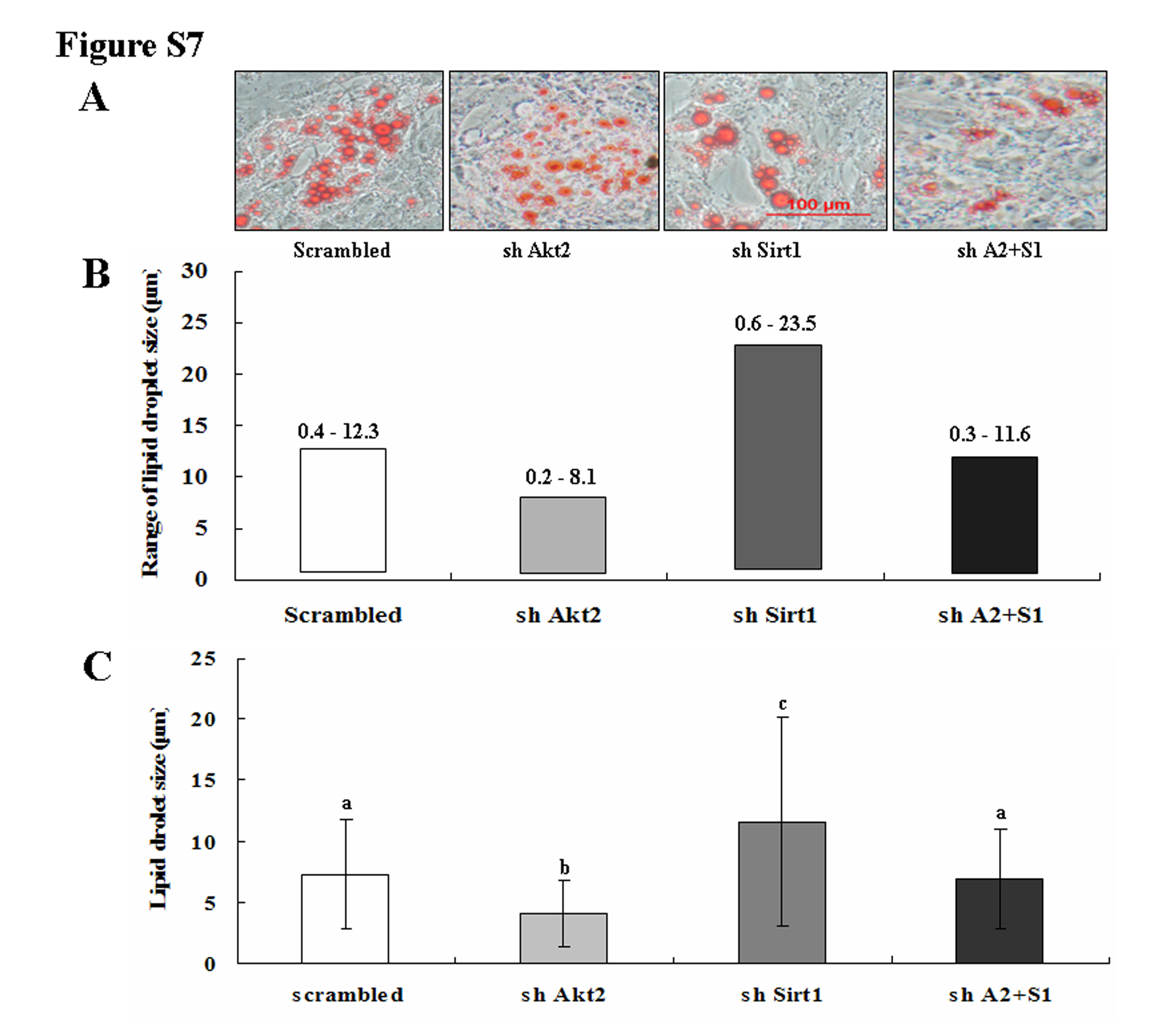

Supplement: Figure S7 — Knockdown of Akt2 and Sirt1 affects lipid droplet size. A. Oil Red O staining at day 8 after infection. Bar, 100 µm or magnification: ×200. B. Range of the lipid droplet size (µm). C. Average value of lipid droplet size (µm). Different letter indicates P<0.05. (TIF) [file pone.0071576.s007.tif]

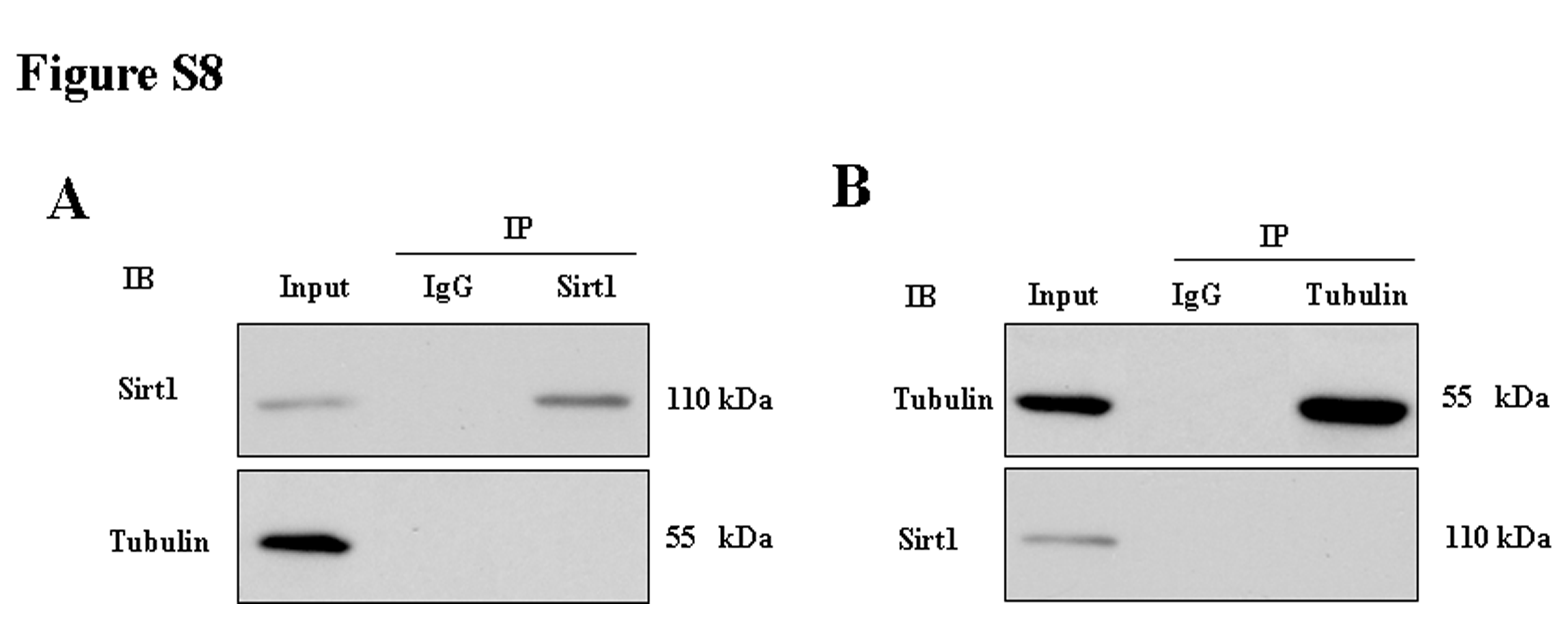

Supplement: Figure S8 — Sirt1 does not interact with Tubulin in adipocytes. At day 8 after induction, porcine fat cells were collected and protein was isolated for immunoprecipitated analysis. A. Sirt1 does not interact with Tubulin. Endogenous Sirt1 in fat cell lysate was immunoprecipitated with anti–Sirt1 antibody, and coprecipitation of Sirt1 was detected by Western blot assay. B. Tubulin does not interact with Sirt1. Endogenous Tubulin in fat cell lysate was immunoprecipitated with anti–Tubulin antibody, and coprecipitation of Tubulin was detected by Western blot assay. (TIF) [file pone.0071576.s008.tif]
