# Supplementary material for: Sirt1 Inhibits Akt2-Mediated Porcine Adipogenesis Potentially by Direct Protein-Protein Interaction
Source: PLoS One. 2013 Aug 12;8(8):e71576. doi: 10.1371/journal.pone.0071576 (PMC3741135; doi:10.1371/journal.pone.0071576)
Supplement: Table S1 — Porcine Akt2 shRNA sense strands and antisense strands. (DOC) [file pone.0071576.s009.doc]

Table S1 Porcine Akt2 shRNA sense strands and antisense strands

| Akt2 shRNAs | Sense strands | Antisense strands |
| --- | --- | --- |
| shRNA1 | 5’–TCGAGAAAAAGGTTCTTCCTCACCGTCAACTCTCGAGAGTTGACGGTGAGGAAGAACCG–3’ | 5’–GATCCGATCTTTCATTGGGTATAAGGCTCGAGCCTTATACCCAATGAAAGATCTTTTTC–3’ |
| shRNA2 | 5’–TCGAGAAAAAGATCTTTCATTGGGTATAAGGCTCGAGCCTTATACCCAATGAAAGATCG–3’ | 5’–GATCCGGGCCAAAGTGACCATGAATGCTCGAGCATTCATGGTCACTTTGGCCCTTTTTC–3’ |
| shRNA3 | 5’–GATCCGCTAGAGGACAATGACTATGGCTCGAGCCATAGTCATTGTCCTCTAGCTTTTTC–3’ | 5’–TCGAGAAAAAGCTAGAGGACAATGACTATGGCTCGAGCCATAGTCATTGTCCTCTAGCG–3’ |
| Scrambled | 5’–GATCCGACACCTACGCAAAACCCTCTCGAGAGGGTTTTGCGTAGGTGTCTTTTTC–3 | 5’–TCGAGAAAAAGACACCTACGCAAAACCCTCTCGAGAGGGTTTTGCGTAGGTGTCG–3’ |
