# Supplementary material for: Sirt1 Inhibits Akt2-Mediated Porcine Adipogenesis Potentially by Direct Protein-Protein Interaction
Source: PLoS One. 2013 Aug 12;8(8):e71576. doi: 10.1371/journal.pone.0071576 (PMC3741135; doi:10.1371/journal.pone.0071576)
Supplement: Table S2 — Porcine Sirt1 shRNAs sense strands and antisense strands. (DOC) [file pone.0071576.s010.doc]

Table S2 Porcine Sirt1 shRNAs sense strands and antisense strands

| Sirt1 shRNAs | Sense strands | Antisense strands |
| --- | --- | --- |
| shRNA1 | 5’–GATCCGCCATCTCTCTGTCACAAATTCAAGAGATTTGTGACAGAGAGATGGTTTTTTC–3’ | 5’–TCGAGAAAAAACCATCTCTCTGTCACAAATCTCTTGAATTTGTGACAGAGAGATGGCG–3’ |
| shRNA2 | 5’–GATCCGCCAGTAGCACTAATTCCAATCAAGAGTTGGAATTAGTGCTACTGGTTTTTTC–3’ | 5’–TCGAGAAAAAACCAGTAGCACTAATTCCAACTCTTGATTGGAATTAGTGCTACTGGCG–3’ |
| shRNA3 | 5’–GATCCGGATGAAAGTGAGATTGAATCAAGAGTTCAATCTCACTTTCATCCTTTTTTC–3’ | 5’–TCGAGAAAAAAGGATGAAAGTGAGATTGA ACTCTTGATT CAATCTCACTTTCATCCG–3’ |
| Scrambled | 5’–GATCCGACACCTACGCAAAACCCTCTCGAGAGGGTTTTGCGTAGGTGTCTTTTTC–3 | 5’–TCGAGAAAAAGACACCTACGCAAAACCCTCTCGAGAGGGTTTTGCGTAGGTGTCG–3’ |
